# Landmark Cancer Clinical Trials and Real-World Patient Populations: Examining Race and Age Reporting

**DOI:** 10.3390/cancers13225770

**Published:** 2021-11-18

**Authors:** Thejus Jayakrishnan, Sonikpreet Aulakh, Mizba Baksh, Kianna Nguyen, Meghna Ailawadhi, Ayesha Samreen, Ricardo Parrondo, Taimur Sher, Vivek Roy, Rami Manochakian, Aneel Paulus, Asher Chanan-Khan, Sikander Ailawadhi

**Affiliations:** 1 Department of Hematology and Medical Oncology, Taussig Cancer Institute, Cleveland, OH 44106, USA; thayyit@ccf.org; 2 Departments of Internal Medicine and Neurosciences, West Virginia University, Morgantown, WV 26506, USA; sonikpreet.aulakh@hsc.wvu.edu; 3 Division of Hematology-Oncology, Mayo Clinic, Jacksonville, FL 100151, USA; baksh.mizba@mayo.edu (M.B.); ailawadhi.meghna@mayo.edu (M.A.); asamreen@huhosp.org (A.S.); parrondo.ricardo@mayo.edu (R.P.); sher.taimur@mayo.edu (T.S.); Roy.Vivek@mayo.edu (V.R.); manochakian.rami@mayo.edu (R.M.); chanan-khan.asher@mayo.edu (A.C.-K.); 4 Mayo Clinic Alix School of Medicine, Rochester, MN 55905, USA; nguyen.kianna@mayo.edu; 5 Division of Cancer Biology, Mayo Clinic, Jacksonville, FL 32224, USA; Paulus.Aneel@mayo.edu; 6 Division of Hematology-Oncology, St. Vincent’s Cancer Center, Jacksonville, FL 32224, USA

**Keywords:** clinical trials, cancer, disparity, age, race

## Abstract

**Simple Summary:**

Food and Drug Administration (FDA) drug approvals from July 2007 to June 2019 were reviewed to identify oncology approvals, and trials with age details were reviewed for the study. We hypothesized that the clinical trials that do not report race are likely to suffer from a higher degree of age disparity. The study demonstrated that a significant number of clinical trials leading to cancer drug approvals suffer from racial and age disparity when compared to real-world populations and that the two factors may be interrelated. Age discrepancy between the clinical trial population and the real-world population was higher for studies that did not report race (mean difference −8.8 years (95% CI −12.6 to −5.0 years)) vs. studies that did report it. We recommend continued efforts to recruit diverse populations in clinical trials and make concerted efforts to implement national strategies in order to realize healthcare equity. In the meantime, detailed reporting of patient demographic characteristics in publications should be considered standard.

**Abstract:**

Background: Concern exists that the clinical trial populations differ from respective cancer populations in terms of their age distribution affecting the generalizability of the results, especially in underrepresented minorities. We hypothesized that the clinical trials that do not report race are likely to suffer from a higher degree of age disparity. Methods: Food and Drug Administration (FDA) drug approvals from July 2007 to June 2019 were reviewed to identify oncology approvals, and trials with age details were selected. The outcomes studied were the weighted mean difference in age between the clinical trial population and real-world population for various cancers, the prevalence of race reporting and association of age and race reporting with each other. Results: Of the 261 trials, race was reported in 223 (85.4%) of the trials, while 38 trials (14.6%) had no mention of race. Race reporting improved minimally over time: 29 (85.3%) in 2007–2010 vs. 49 (80.3%) in 2011–2014 vs. 145 (85.4%) during the period 2015–2019 (*p*-value = 0.41). Age discrepancy between the clinical trial population and the real-world population was higher for studies that did not report race (mean difference −8.8 years (95% CI −12.6 to −5.0 years)) vs. studies that did report it (mean difference −5.1 years, (95% CI −6.4 to −3.7 years), *p*-value = 0.04). Conclusion: The study demonstrates that a significant number of clinical trials leading to cancer drug approvals suffer from racial and age disparity when compared to real-world populations, and that the two factors may be interrelated. We recommend continued efforts to recruit diverse populations.

## 1. Introduction

The unfortunate reality of the lack of representation of racial and ethnic minorities in clinical trials is a major barrier to the achievement of health equity [1,2,3,4,5]. While this is of importance to all aspects of medicine, the relevance in the context of cancer stems from the fact that the cancer treatment is life-altering, and differences based on the race and ethnic characteristics of the patient have implications on survival and quality of life. It is therefore concerning that the reporting of race has not improved over the years to adequately monitor minority representation, and certainly, the clinical trials that do not report race may be not capturing a diverse population, representative of the true U.S. demographic [1]. Furthermore, certain cancer diagnoses are seen more frequently in some racial/ethnic subgroups, making this under-representation even more challenging [6]. Another factor that represents an important baseline characteristic of the trial population, with implications on the applicability of the trial results in the real-world setting, is patient age [2]. Moreover, the interaction of race/ethnicity and patient age is important as age patterns for cancers can be unique to certain races, making this especially relevant for clinical trials leading to drug approval or label changes. Despite this, it has been shown previously that racial minorities and certain age-groups, especially the elderly, are under-represented in clinical trials [7].

We aimed to analyze age and race reporting in landmark cancer clinical trials leading to drug approvals. We hypothesized that the clinical trials that do not report data on patients’ race do not adequately represent the US population demographics and, as such, are more likely to omit other vulnerable patient groups, especially the elderly. Therefore, a comprehensive analysis of these clinical trials will demonstrate discordance for age compared to the real-world population and this can significantly impact the applicability and generalizability of trial results.

## 2. Methods

### 2.1. Data Collection and Outcomes

We reviewed Food and Drug Administration (FDA) drug approvals from July 2007 through June 2019 and identified drug approvals specifically pertaining to malignant hematology and oncology. Trial details were then reviewed using the following sources: FDA website, National Institutes of Health (NIH) trials registry (ClinicalTrials.gov), publications available online, and online package drug inserts to extensively cross-examine available clinical trial information resources regarding patient age and race/ethnicity representation. The trials were then screened using the pre-specified eligibility criteria for inclusion in the study. The study did not involve any patient-identifying information and therefore was exempt from institutional review board approval.

Inclusion and exclusion criteria used were as follows:

1. Inclusion criteria: age details available for the trial at any of the above sources.
2. Exclusion criteria: age details not available, non-malignant hematological conditions, drug approvals for cancer of multiple sites, or pediatric cancers.

The characteristics of the trial were then extracted. When multiple trials on the same drug were available, they were extracted separately provided they met the above eligibility criteria. The average age of cancer diagnosis for various cancers was obtained using data published at cancer.org and seer.cancer.org (accessed on 1 August 2020) [8,9].

The primary outcome was the weighted mean difference in age (in years) between the clinical trial population and the real-world population of cancer patients. The age difference was obtained for each trial and combined for each cancer studied (please see the statistical analysis section for more details). The secondary outcomes were prevalence of race reporting and association of age and race reporting with each other, as well as other trial characteristics—namely cancer studied, type of cancer (solid versus hematological), phase of the trial (1/2 vs. 3 vs. 4), type of drug approval (change of label or new indication), and year of approval.

### 2.2. Statistical Analysis

Categorical variables are described as percentages and continuous variables as median (IQR = interquartile range) or mean (95% confidence interval) as applicable.

To calculate the difference in age between the clinical trial population and real-world population, we first extracted the age details available from various sources as listed above. Mean and median ages were both included with preference to mean ages where both were available. These were then used to calculate age difference (age from the trial minus the age of the real-world cancer population) and the weighted mean for the age difference for each type of cancer (weighted by the sample size of the studies).

Group comparisons for proportions were performed using the χ^2^ test. The association of the discrepancy in age (between clinical trial age and real-world age) across different cancers was performed using Student’s *t*-test. Similarly, the impact of trial characteristics—namely the type of cancer (solid versus hematological), race reporting, phase of the trial (1/2 vs. 3 vs. 4), type of drug approval (change of label or new indication), year of approval—were assessed by a multiple analysis of variance (MANOVA) test or Student’s *t*-test (depending on the number of levels for each variable) using weighted age difference as the dependent factor and the other variables as independent variables. *p*-values were 2-sided and considered statistically significant when unadjusted *p* < 0.05. The analysis was performed using STATA version 16.

## 3. Results

**Figure 1:**
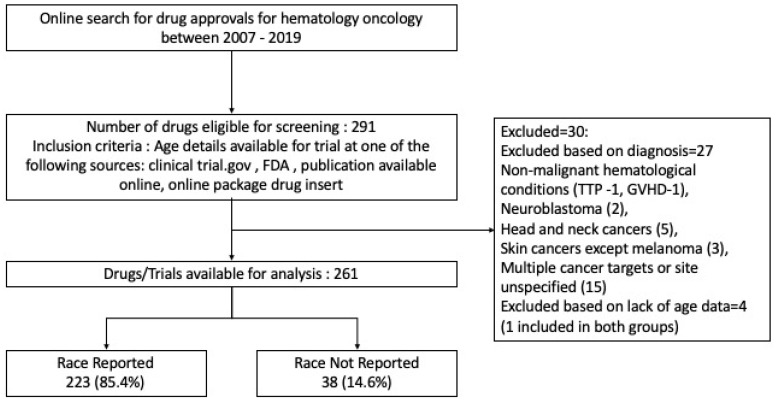
CONSORT Outlining the selection process for the study.

The selection process for the clinical trials included in the study is outlined in Figure 1. On initial screening, 291 drug trials were identified and of these, 261 trials were selected for final analysis after applying the eligibility criteria described above.

The average age for patients in the selected trials was 61 years (IQR 55-64) with an average sample size of 405 (185–707). The majority of the trials were in the year group 2015–2019 (166 trials; 63.6%) and included drug approvals representing new indications (208 trials; 79.7%). Similarly, most were phase 3 (168 trials; 64.4%) and randomized (200; 76.6%). Baseline characteristics of the selected trials are outlined in Table 1.

### 3.1. Race Disparity

**Figure 2:**
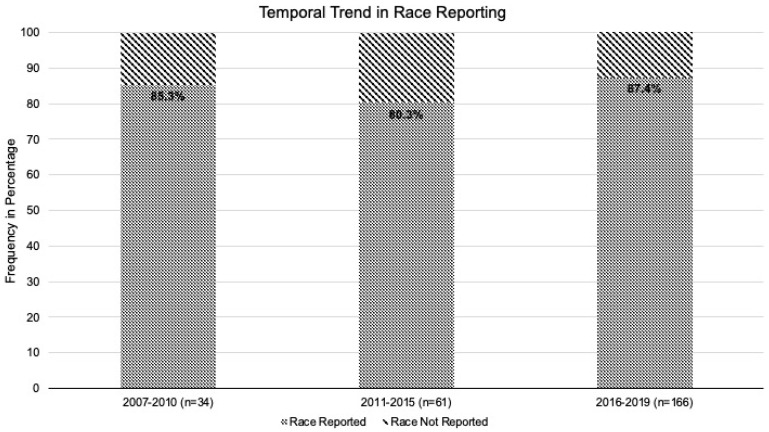
Trends in race reporting over time in clinical trials leading to cancer drug approval between 2007 and 2019.

Of the 261 trials, race was reported in 223 (85.4%) trials while 38 trials (14.6%) had no mention of it. Trials without any race reporting included 10 changes of the label and 28 with new drug indication approvals. Of these 38 trials without race reporting, 13 (14.2%) were phase 1/2 and 25 (14.9%) were phase 3. Among the trials selected for final analysis, there were only two phase 4 trials and both reported patient racial status. Race reporting was not impacted by the type of trial (change of label versus new indication; *p*-value = 0.32) or the phase of the trial (*p*-value = 0.84). There was no significant association between race reporting and whether trials were for solid organ (147 trials reported race; 86.5%) or hematologic cancer (76 trials reported race; 83.5%) diagnosis (*p*-value = 0.52). Notably, race reporting did not improve significantly over time among landmark cancer clinical trials (29 trials; 85.3% in 2007–2010 vs. 49 trials; 80.3% in 2011–2014 vs. 145 trials; 85.4% in 2015–2019) (*p*-value = 0.41, Figure 2).

### 3.2. Age Disparity

**Figure 3:**
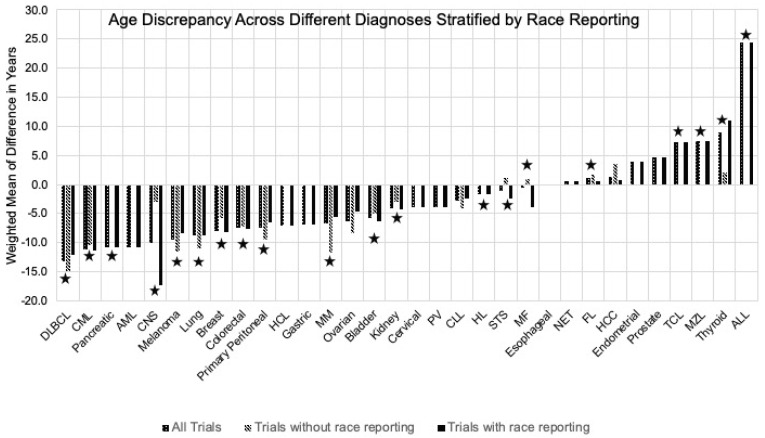
Graphical representation of discrepancy in age between clinical trial populations and real-world patients according to the cancer type and race reporting. Starred groups indicate presence of statistically significant difference.

The discrepancy in ages between the cancer trial population and real-world population (measured as weighted mean difference) is outlined in Table 2 and represented in Figure 3. A negative weighted mean difference indicates the pooled trial population was older on average. On analysis of variance of the age differences by various clinical trial characteristics, no significant association was found with the year of the clinical trial (*p*-value = 0.47), cancer type (solid vs. hematological cancer, *p*-value = 0.31), type of approval (change of label vs. new indications, *p*-value = 0.90), or phase of the trial (*p*-value = 0.36). However, the discrepancy between reported average age of the trial population and the real-world population for that cancer was higher for studies that did not report race (mean difference −8.8 years, 95% CI −12.6 to −5.0 years) vs. studies that did report race (mean difference −5.1 years, 95% CI −6.4 to −3.7 years) (*p*-value = 0.04).

## 4. Discussion

Clinical trials represent the cutting edge of medicine leading to novel therapies. In the context of cancer, they lead to the improvement in overall survival or disease-free survival, ideally at an acceptable quality of life and cost to the patient and society. The need for adequate representation of the diverse patient population in clinical trials cannot be overstated. This is especially relevant in the era of personalized medicine and immunotherapy where tumor response and toxicity could vary based on the individual or population group [4]. However, studies consistently show that the clinical trial population is dissimilar to the real-world population, particularly in the involvement of racial and ethnic minorities, women, and patients with gender incongruence, as well as the elderly [1,2,3,4,5]. Subgroup analyses involving racial subgroups are often underpowered owing to the under-representation of minorities [1,3,27]. This raises questions about the applicability of toxic therapy and predictive biomarkers on diverse population groups [4]. Thus, the present study was carried out to assess the disparity between the population involved in clinical trials and the real world for racial minorities, and to understand the interaction of race and age with this disparity. We demonstrate that a significant number of these crucial trials do not report race, with no improvement over the study period. Moreover, significant differences in patient ages between clinical trials and the real world exist for several cancer groups, particularly in trials where race is not reported, further affecting the applicability and generalizability of clinical trials for racial minorities.

It appears that clinical trials leading to drug approval have not shown improvement in the accrual of minority populations even after the introduction of appropriate policy changes such as the FDA/National Institute of Health (NIH) mandates on proportional racial representation [28]. A study that included clinical trials for the years 2008–2018 also had similar findings to ours [1]. The study population was however different from ours as the current study included more years and had an additional focus on patient age, not just racial disparities. For this purpose, we included only studies that mentioned median and range of age for the patients enrolled. Only about 63% of the 230 trials that were included reported race and remained unchanged over the study period. The study showed that racial minorities were consistently underrepresented in clinical trials, and that minorities enrolled in smaller, non-randomized single-arm trials, raising concerns about the recruitment methods [1]. Moreover, only around 20% of the randomized controlled studies published in high-impact oncology journals reported result by race or ethnicity [28]. Geriatric patients are also under-represented in clinical trials, accounting for 10 to 40% of the clinical trial populations [2]. This may be a result of the stringent inclusion criteria [2]. In the current study we have attempted to show that the disparity in age between the clinical trial population and the real-world population is significant for several cancer groups. The findings also support the hypothesis that disparity in age and race coexists, indicating that factors contributing to these may be interrelated. Furthermore, we noted that the disparity in one of these factors may be heightened when the other pre-exists.

Several reasons can be proposed for the findings. These may include the disparities in access to treatment driven by social determinants of health, or possible distrust of the under-represented minorities towards clinical trials due to historic discrimination [1]. Similarly, patients in the elderly age group can be frequently excluded from clinical trials due to coexisting comorbidities, performance status, or higher dependency on a support system to receive medical care [2]. Interestingly, one study that utilized Behavioral Risk Factor Surveillance System (BRFSS) showed that the clinical trials (not limited to those leading to drug approvals like the current study) did have adequate representation in relation to non-Hispanic blacks but redemonstrated disparities with respect to gender, age and socioeconomic status [29]. The result, that was contradictory to previous studies, was attributed to differences in proportions of racial minorities in both the general population and the cancer population. However, other authors have shown that, even when adjusted for population age and demographic characteristics, racial minorities remain under-represented in clinical trials especially those leading to drug approvals [1]. Thus, the finding from the study mentioned above could be related to the sample that was used and recall bias. Cost of care and rigid timelines/requirements associated with clinical trials can make it onerous to perform study-related procedures and possibly impact minority accrual [30].

Interventions to address clinical trial recruitment disparity should take into consideration the above-mentioned mechanisms. Policies such as an NIH mandate for inclusion of minorities aim to improve racial representation [28,31]. Unfortunately, this has not been successful in showing a positive impact—at least, based on the recent studies suggesting the need for improved efforts or strategies that may help implement the recommendations [28]. This could be because these mandates apply only for NIH-funded research while most trials leading to drug approvals are funded by industry [1]. Typically, pharmaceutical company-sponsored trials have poorer representation of black patients [8]. Even for the National Cancer Institute’s (NCI) clinical trials, less than 2% had a focus on the racial/minority population as their primary emphasis [28]. Other interventions include incorporating enhanced models of patient navigation, and community partnership may be beneficial to overcome this minority accrual disparity [32]. Strategies such as video-based education have also been effective in the clinical trial setting for improving enrollment of minorities [33,34]. Most importantly, we would reiterate the recommendations to report race and age in clinical trial publications to adequately monitor representation [1]. Ultimately, multi-level interventions including comprehensive efforts in addressing the social determinants of health, availability of adequate resources and objective metrics to measure improvement, along with regulatory and advocacy will power, are likely to be effective in improving healthcare equity [30,34].

Our study does have some limitations. An explanation for the lack of improvement in diversity in clinical trials could be the globalization of the trials leading to the trials not mirroring the United States population. It is possible that the racial status was not reported in trials performed outside the United States [35]. In our opinion, this does not justify the issue related to lack of reporting of race, as the approved drugs are utilized globally. To interpret the study, we assumed that the rates of minority enrollment, in studies that did not report race, are low. This has been suggested by others as well but is a conservative approach in assessing racial representation [1]. While we carried out an exhaustive search for race reporting and age information as outlined in the methods, it is possible that we missed some trials that reported them, but the fraction could be expected to be extremely small, thereby not altering the findings of the present study. Lastly, we did not make a direct comparison between real-world racial distribution and racial distribution in trials as that was not the objective of the present study.

## 5. Conclusions

Racial and age disparities with ongoing, significant under-representation of the true U.S. population demographic, in a significant number of landmark studies leading to cancer drug approvals, are a major concern. These can potentially risk realizing the true benefits and risks of approved therapeutic strategies, and their real-world impact. We recommend continued efforts to recruit diverse populations in clinical trials and make concerted efforts to implement national strategies in order to realize healthcare equity. In the meantime, detailed reporting of patient demographic characteristics in publications should be considered standard.

## Figures and Tables

**Table 1:** Baseline characteristics of trials included in the analysis.

Characteristic	Number of Trials (%) or Median (IQR Interquartile Range)
Age (years)	61 (55–64)
Sample size	405 (185–707)
Year of approval	
2007–2010	34 (13.0%)
2011–2014	61 (23.4%)
2015–2019	166 (63.6%)
Type of Approval	
New Indication	208 (79.7%)
Change of Label	53 (20.3%)
Phase of Trial	
Phase 1/2	91 (34.8%)
Phase 3	168 (64.4%)
Phase 4	2 (0.8%)
Randomized	
Yes	200 (76.6%)
No	61 (23.4%)
Disease Type	
Hematological	
Acute	
Acute Myeloid Leukemia	8 (3.1%)
Acute Lymphoblastic Leukemia	4 (1.5%)
Chronic	
Chronic Lymphocytic Leukemia	16 (6.1%)
Chronic Myeloid Leukemia	11 (4.2%)
Myelofibrosis	2 (0.8%)
Polycythemia Vera	1 (0.4%)
Other	
Diffuse Large B-Cell Lymphoma	8 (3.1%)
Follicular Lymphoma	6 (2.3%)
T-Cell Lymphoma	7 (2.7%)
Marginal Zone Lymphoma	5 (1.9%)
Hodgkin Lymphoma	5 (1.9%)
Multiple Myeloma	17 (6.5%)
Hairy Cell Leukemia	1 (0.4%)
Solid	
Lung	37 (14.2%)
Breast	26 (10.0%)
Melanoma	17 (6.5%)
Kidney	15 (5.8%)
Prostate	11 (4.2%)
Colorectal	9 (3.5%)
Primary Peritoneal	9 (3.5%)
Hepatocellular carcinoma	6 (2.3%)
Bladder	6 (2.3%)
Soft Tissue Sarcoma	6 (2.3%)
Gastric	5 (1.9%)
Pancreatic	5 (1.9%)
Thyroid	5 (1.9%)
Ovarian	4 (1.5%)
Central Nervous System	3 (1.2%)
Esophageal	2 (0.8%)
Neuroendocrine Tumor	2 (0.8%)
Endometrial	1 (0.4%)
Cervical cancer	1 (0.4%)

**Table 2:** Outlining the discrepancy in age between the trial population and actual population.

Disease Type	Average Age in Years (Population)	Sample Size (Combined for All Trials)	Weighted Mean Difference of Ages ^a^; Mean (IQR)	*p*-Value	Reference
Hematological					
Acute					
Acute Myeloid Leukemia	68	2361	−10.8 (−21.0 to −1.0)	0.156	[10]
Acute Lymphoblastic Leukemia	14	954	24.3 (23.0 to 33.0)	0.039	[11]
Chronic					
Chronic Myeloid Leukemia	64	4512	−11.2 (−11.0 to −5.0)	0.006	[11]
Chronic Lymphocytic Leukemia	71	5152	−2.9 (−1.0 to −6.0)	0.039	[11]
Other					
Hodgkin Lymphoma	38	2286	−2.0 (−2.0 to −1.7)	0.935	[11]
Follicular Lymphoma	60	3770	1.1 (−1.0 to 3.0)	0.019	[8]
Diffuse Large B-Cell Lymphoma	70	1473	−13.2 (−14.0 to −4.0)	0.002	[12]
Hairy Cell Leukemia	67	80	−7.0 (no IQR)		[11]
Multiple Myeloma	70	8683	−6.7 (−8.6 to −4.0)	0.0005	[13]
T-Cell Lymphoma	53	1421	7.2 (5.0 to 11.0)	0.0001	[14]
Marginal Zone Lymphoma	60	543	7.4 (7.0 to 8.0)	0.007	[15]
Myelofibrosis	64	420	−0.6 (−4.0 to 1.0)	0.609	[11]
Solid					
Pancreatic	71	1454	−10.8 (−13.0 to −8.0)	<0.005	[8]
Central Nervous System	57	169	−10.1 (no IQR)	0.293	[11]
Melanoma	65	10,650	−9.5 (−14.0 to −4.0)	<0.005	[16]
Lung	70	19,804	−8.7 (−9.0 to −6.0)	<0.005	[17]
Ovarian	63	687	−6.4 (−5.0 to −3.0)	0.130	[11]
Breast	62	24,739	−8.0 (−11.0 to −6.5)	<0.005	[8]
Colorectal	69	6145	−7.5 (−8.0 to −6.0)	<0.005	[11]
Gastric	68	2380	−6.9 (−8.0 to −6.0)	<0.005	[18]
Primary Peritoneal Carcinoma	67	4182	−7.5 (−11.0 to −6.0)	<0.005	[19]
Bladder	73	1440	−5.8 (−7.0 to −5.0)	<0.005	[20]
Cervical	49	98	−4.0 (no IQR)	n/a	[11]
Kidney	64	9099	−4.2 (−5.0 to −2.0)	0.009	[21]
Soft Tissue Sarcoma	58	2892	−1.1 (−3.0 to 0.5)	0.301	[11]
Esophageal	65	749	0 (IQR)	n/a	[22]
Neuroendocrine Tumor	63	433	0.5 (0.0 to 1.0)	0.423	[23]
Hepatocellular Carcinoma	62	3232	1.2 (0.0 to 2.0)	0.053	[24]
Endometrial	60	54	4.0 (IQR)	n/a	[25]
Prostate	66	12,375	4.6 (3.0 to 8.0)	<0.005	[26]
Thyroid	50	1496	9.0 (5.0 to 13.0)	0.014	[11]

^a^ Weighted by sample size.

## Data Availability

Authors T.J. (Thejus Jayakrishnan) and S.A. (Sikander Ailawadhi) had full access to all the data in the study. We take full responsibility for the integrity of the data and the accuracy of the analysis as well as sharing the data with any interested investigators. The datasets used and/or analyzed during the current study are available from the corresponding author on reasonable request.
